# Predictive Performance of the STOP-BANG Questionnaire for Difficult Airway Management in Iraqi Adults Undergoing Elective Surgery: A Single-Center Prospective Observational Cohort Study

**DOI:** 10.7759/cureus.112428

**Published:** 2026-07-10

**Authors:** Sajjad Alshtait, Leila Emami, Faranak Behnaz

**Affiliations:** 1 Department of Anesthesia, Nasiriyah Teaching Hospital, Dhi Qar, IRQ; 2 School of Medicine, Shahid Beheshti University of Medical Sciences, Tehran, IRN; 3 Department of Anesthesiology, Shahid Beheshti University of Medical Sciences, Tehran, IRN

**Keywords:** airway management, difficult airway, difficult intubation, difficult mask ventilation, elective surgery, obstructive sleep apnea, perioperative assessment, preoperative screening, risk stratification, stop-bang

## Abstract

Background: Failure to anticipate airway difficulty may increase the risk of major perioperative complications during anesthesia. The STOP-BANG questionnaire is a simple screening tool for obstructive sleep apnea (OSA) that may also help identify patients at risk of difficult airway management. Evidence from Iraqi surgical populations remains limited.

Objective: This study aimed to evaluate the predictive performance of the STOP-BANG questionnaire for difficult airway management in Iraqi adult patients undergoing elective surgery under general anesthesia.

Methods: This single-center prospective observational cohort study included 500 adults aged 18 to 70 years undergoing elective surgery under general anesthesia at Nasiriyah Teaching Hospital, Dhi Qar Governorate, Iraq. The STOP-BANG questionnaire was completed preoperatively. The primary composite outcome was any airway difficulty, defined as difficult mask ventilation and/or difficult intubation. Receiver operating characteristic analysis was used to assess discrimination. Risk ratios were calculated for the primary cohort comparison, and multivariable logistic regression was used to estimate adjusted odds ratios.

Results: Difficult intubation and difficult mask ventilation occurred in 213 (42.6%) and 80 (16.0%) patients, respectively. The primary composite outcome of any airway difficulty occurred in 218 (43.6%) patients. A STOP-BANG score of ≥5 identified 175 (35.0%) high-risk patients. The risk of any airway difficulty was 146/175 (83.4%) among patients with STOP-BANG ≥5 and 72/325 (22.2%) among patients with STOP-BANG <5, corresponding to an unadjusted risk ratio of 3.77 (95% CI, 3.04-4.66). For the primary composite outcome, STOP-BANG ≥5 demonstrated a sensitivity of 146/218 (66.97%), specificity of 253/282 (89.72%), positive predictive value of 146/175 (83.43%), and negative predictive value of 253/325 (77.85%), with an area under the receiver operating characteristic curve of 0.783. The continuous STOP-BANG score showed greater discriminative ability, with an area under the curve of 0.870. In multivariable analysis, STOP-BANG ≥5 remained independently associated with airway difficulty and difficult intubation.

Conclusions: The STOP-BANG questionnaire showed clinically useful discrimination for difficult airway management among Iraqi adults undergoing elective surgery under general anesthesia. These findings support considering STOP-BANG as a practical adjunctive preoperative risk-stratification tool that complements, but does not replace, comprehensive airway assessment and clinical judgment. Further multicenter external validation is needed before broad implementation.

## Introduction

Failure to predict difficult airways increases the risk of hypoxemia, aspiration, hemodynamic instability, and other perioperative complications during anesthesia [1,2]. Therefore, effective preoperative risk stratification is an important component of safe airway management, particularly in patients with obesity or suspected obstructive sleep apnea (OSA) [3,4].

OSA has been associated with difficult tracheal intubation, difficult mask ventilation, and increased perioperative respiratory complications [5-7]. The STOP-BANG questionnaire is a practical eight-item screening tool that is widely used in perioperative settings to identify patients at increased risk of OSA [4,8-12]. Previous systematic reviews have shown that STOP-BANG has strong screening performance for OSA across diverse populations and may provide clinically useful information when assessing airway-related risk [8-10].

Evidence regarding the use of STOP-BANG for predicting difficult airway management in Middle Eastern surgical populations remains limited. The primary objective of this study was to evaluate the predictive performance of the STOP-BANG questionnaire for any airway difficulty among Iraqi adults undergoing elective surgery under general anesthesia. The primary endpoint was any airway difficulty, defined as difficult mask ventilation and/or difficult intubation. Secondary analyses examined difficult intubation and difficult mask ventilation individually, as well as the association between STOP-BANG ≥5 and airway outcomes. We hypothesized that higher STOP-BANG scores would be associated with increased risk of airway difficulty and would provide clinically useful discrimination as an adjunct to conventional preoperative airway assessment.

## Materials and methods

Study design and setting

This single-center prospective observational cohort study was conducted at Nasiriyah Teaching Hospital, Dhi Qar Governorate, Iraq, from November 2025 to March 2026. Ethical approval was obtained from the Research Ethics Committee, School of Medicine, Shahid Beheshti University of Medical Sciences, Tehran, Iran (approval no. IR.SBMU.MSP.REC.1404.482; approved on November 5, 2025). Written informed consent was obtained from all participants before enrollment.

Participants

Adults aged 18 to 70 years who were scheduled for elective surgery under general anesthesia were eligible for inclusion. Patients with a documented history of difficult intubation or ventilation, severe respiratory disease, including chronic obstructive pulmonary disease, critical cardiovascular disease, or neuromuscular disorders, were excluded.

A consecutive convenience sample of 500 eligible patients who met the inclusion criteria and had no exclusion criteria was enrolled and included in the final analysis. Patients who did not meet the eligibility criteria were not enrolled, and a separate screening log of non-enrolled patients was not maintained.

Study objectives, endpoint, and performance measures

The primary objective of this study was to evaluate the predictive performance of the preoperative STOP-BANG questionnaire for identifying the primary composite endpoint of any airway difficulty. The primary endpoint was the composite outcome of difficult mask ventilation and/or difficult intubation. The primary performance measure was the area under the receiver operating characteristic curve for the continuous STOP-BANG score. Secondary performance measures included sensitivity, specificity, positive predictive value, and negative predictive value for the prespecified STOP-BANG cutoff of ≥5, as well as secondary analyses of difficult intubation and difficult mask ventilation separately. Multivariable logistic regression was used to evaluate adjusted associations between STOP-BANG ≥5 and airway outcomes. The primary hypothesis was that higher STOP-BANG scores would demonstrate clinically useful discrimination for any airway difficulty and that STOP-BANG ≥5 would remain associated with airway difficulty after adjustment for clinically relevant covariates.

STOP-BANG assessment

The STOP-BANG questionnaire was completed in the pre-anesthesia clinic on the day of surgery or up to one week before surgery. Height, weight, and neck circumference were measured directly. Responses regarding snoring, daytime tiredness, and observed apnea were obtained directly from participants. Each positive item was assigned one point, resulting in a total score ranging from 0 to 8. For binary analysis, patients with scores ≥5 were classified as high risk [4,11,12].

The STOP-BANG blood pressure item was considered positive if the patient had a previous physician diagnosis of hypertension, was receiving antihypertensive medication, or had a preoperative blood pressure measurement of ≥140/90 mmHg. This operational definition was selected to align the STOP-BANG pressure item, which asks about high blood pressure or treatment for high blood pressure, with a widely used clinical threshold for hypertension [11,13]. The STOP-BANG questionnaire items used in this study are provided in the Appendix.

Airway outcomes and definitions

Intraoperative airway management was performed using direct laryngoscopy with a Macintosh blade after standard intravenous induction of general anesthesia and neuromuscular blockade with rocuronium. Rapid sequence induction was not part of the routine study protocol. A stylet was used when clinically required. Intraoperative airway assessment was performed and confirmed by anesthesiology specialists. Experienced anesthesia technicians assisted in airway management and documentation when required, but final airway outcome assessment was based on the anesthesiology specialist’s evaluation according to prespecified definitions. A prespecified data collection form and standardized outcome definitions were used to reduce variability in assessment.

Difficult mask ventilation was defined as Han grade III or IV [14]. Difficult intubation was defined as an Intubation Difficulty Scale score ≥5, three or more intubation attempts, or Cormack-Lehane grade III or IV [15,16]. The primary composite endpoint was any airway difficulty, defined as difficult mask ventilation and/or difficult intubation. Mallampati classification, thyromental distance, and upper lip bite test were evaluated as preoperative airway assessment tools [17,18]. Cormack-Lehane grade was retained only as an intraoperative component of the difficult-intubation definition and was not evaluated as an independent preoperative predictor.

Intraoperative assessors were not provided with STOP-BANG scores before airway assessment. The study was conducted in a real-world single-center clinical setting, and complete separation between preoperative and intraoperative clinical teams could not always be guaranteed. This issue is addressed as a potential limitation of ascertainment bias and interobserver variability.

Statistical analysis

Continuous variables were summarized as mean ± standard deviation, and categorical variables were summarized as frequencies and percentages. Pearson correlation analysis was used to evaluate associations between STOP-BANG scores and airway outcomes. Receiver operating characteristic curve analysis was performed for the continuous STOP-BANG score and for the prespecified cutoff of ≥5. The area under the receiver operating characteristic curve was reported with 95% confidence intervals estimated using nonparametric bootstrap resampling with 2,000 replicates. ROC curves were not formally compared using pairwise statistical testing; comparisons between tools were interpreted descriptively. Sensitivity, specificity, positive predictive value, and negative predictive value were calculated for STOP-BANG ≥5, with 95% confidence intervals estimated using the Wilson method. 

Because this was a prospective cohort design, the unadjusted risk ratio with 95% confidence interval was calculated for the primary composite endpoint by comparing patients with STOP-BANG ≥5 with those with STOP-BANG <5. Multivariable logistic regression was then used to estimate adjusted odds ratios for the association between STOP-BANG ≥5 and airway outcomes after adjustment for clinically relevant covariates.

Multivariable logistic regression models were used to evaluate the independent association between STOP-BANG ≥5 and airway difficulty. Covariates were selected a priori based on clinical relevance and overlap with established difficult-airway risk factors rather than by automated stepwise selection. Covariates included age >50 years, male sex, neck circumference >40 cm, body mass index ≥30 kg/m², and hypertension. Potential multicollinearity was assessed using the variance inflation factor, with values >5 considered suggestive of clinically relevant multicollinearity. Because all regression covariates were binary, the linearity of continuous predictors in the logit was not applicable. Model calibration was assessed internally using the Brier score and the Hosmer-Lemeshow goodness-of-fit test [19]. Missing data were assessed before analysis; because all 500 enrolled patients had complete data for the variables included in the final analyses, complete-case analysis included the full cohort. The number of events for the primary composite endpoint was also considered adequate for the planned multivariable model relative to the number of covariates [20].

Sample size and precision justification

A formal a priori sample size calculation was not performed before study initiation; therefore, the sample size should be interpreted as pragmatic and based on the number of eligible patients available during the study period. To evaluate the adequacy of the enrolled sample for diagnostic-accuracy estimation, a precision-based justification was applied using assumptions relevant to the primary composite endpoint [21,22]. Assuming an expected prevalence of any airway difficulty of approximately 40%, an expected sensitivity of 0.67, an expected specificity of 0.90 for the prespecified STOP-BANG cutoff of ≥5, a two-sided 95% confidence level, and clinically acceptable absolute precision of 0.07 for sensitivity and 0.04 for specificity, the minimum required total sample size would be approximately 434 patients for sensitivity estimation and approximately 361 patients for specificity estimation. Therefore, the enrolled sample of 500 patients was considered adequate for estimating the diagnostic performance of STOP-BANG with clinically acceptable precision. Nevertheless, the absence of a formal a priori sample size calculation is acknowledged as a limitation.

## Results

Baseline characteristics

The final analysis included 500 eligible participants. The study cohort included 273 females (54.6%) and 227 males (45.4%). The mean age was 46.8 ± 11.5 years, the mean body mass index was 28.1 ± 6.3 kg/m², and the mean neck circumference was 37.0 ± 4.0 cm. Hypertension was present in 195 patients (39.0%), diabetes mellitus in 142 patients (28.4%), and preexisting OSA was reported in 98 patients (19.6%). The baseline demographic and clinical characteristics are summarized in Table 1.

**Table 1:** Baseline demographic and clinical characteristics. BMI: body mass index; OSA: obstructive sleep apnea; SD: standard deviation

Characteristic	Total (n = 500)	Male (n = 227)	Female (n = 273)
Age, years (mean ± SD)	46.8 ± 11.5	47.1 ± 11.9	46.5 ± 11.3
BMI, kg/m² (mean ± SD)	28.1 ± 6.3	28.5 ± 6.0	27.8 ± 6.5
Neck circumference, cm (mean ± SD)	37.0 ± 4.0	38.0 ± 3.4	36.2 ± 4.2
Hypertension, n (%)	195 (39.0%)	95 (41.9%)	100 (36.6%)
Diabetes mellitus, n (%)	142 (28.4%)	67 (29.5%)	75 (27.5%)
OSA diagnosis, n (%)	98 (19.6%)	51 (22.5%)	47 (17.2%)

STOP-BANG distribution and airway event rates

STOP-BANG scores were classified as low risk (0-2) in 181 (36.2%) patients, moderate risk (3-4) in 144 (28.8%) patients, and high risk (≥5) in 175 (35.0%) patients. The proportion of patients with STOP-BANG ≥5 did not differ significantly between women and men: 100/273 (36.63%) versus 75/227 (33.04%), respectively; p = 0.46.

Difficult intubation occurred in 213 (42.6%) patients using a composite definition that included IDS ≥5, ≥3 intubation attempts, or Cormack-Lehane grade III/IV. The individual difficult-intubation components were as follows: IDS ≥5 was present in 96 (19.2%) patients, ≥3 intubation attempts occurred in 46 (9.2%) patients, and Cormack-Lehane grade III/IV was observed in 169 (33.8%) patients. These components were not mutually exclusive. Difficult mask ventilation occurred in 80 (16.0%) patients, and the primary composite endpoint of any airway difficulty occurred in 218 (43.6%) patients.

Predictive performance

The continuous STOP-BANG score showed a statistically significant positive correlation with difficult intubation (r = 0.621, p < 0.001), difficult mask ventilation (r = 0.600, p < 0.001), and the primary composite endpoint of any airway difficulty (r = 0.637, p < 0.001). For the primary composite endpoint, the continuous STOP-BANG score demonstrated an AUC of 0.870 (bootstrap 95% CI, 0.838-0.899).

The risk of any airway difficulty was 146/175 (83.4%) among patients with STOP-BANG ≥5 and 72/325 (22.2%) among patients with STOP-BANG <5, corresponding to an unadjusted risk ratio of 3.77 (95% CI, 3.04-4.66).

For difficult intubation alone, the continuous STOP-BANG score demonstrated an AUC of 0.859 (bootstrap 95% CI, 0.826-0.890). Comparative predictive performance of STOP-BANG and preoperative airway assessment tools is summarized in Table 2.

**Table 2:** Comparative predictive performance of STOP-BANG and preoperative airway. AUC: area under the receiver operating characteristic curve. STOP-BANG was evaluated as both a continuous score and a prespecified cutoff of ≥5. Cormack-Lehane grade was not included as a comparator because it was an intraoperative component of the difficult-intubation definition rather than an independent preoperative predictor.

Assessment tool	Sensitivity, n/N (%)	Specificity, n/N (%)	AUC
STOP-BANG (continuous 0-8)	—	—	0.870
STOP-BANG (≥5)	146/218 (66.97%)	253/282 (89.72%)	0.783
Mallampati (≥III)	115/218 (52.75%)	208/282 (73.76%)	0.633
Thyromental distance (<6 cm)	80/218 (36.70%)	209/282 (74.11%)	0.554
Upper lip bite test (Class III)	76/218 (34.86%)	214/282 (75.89%)	0.554

**Figure 1:**
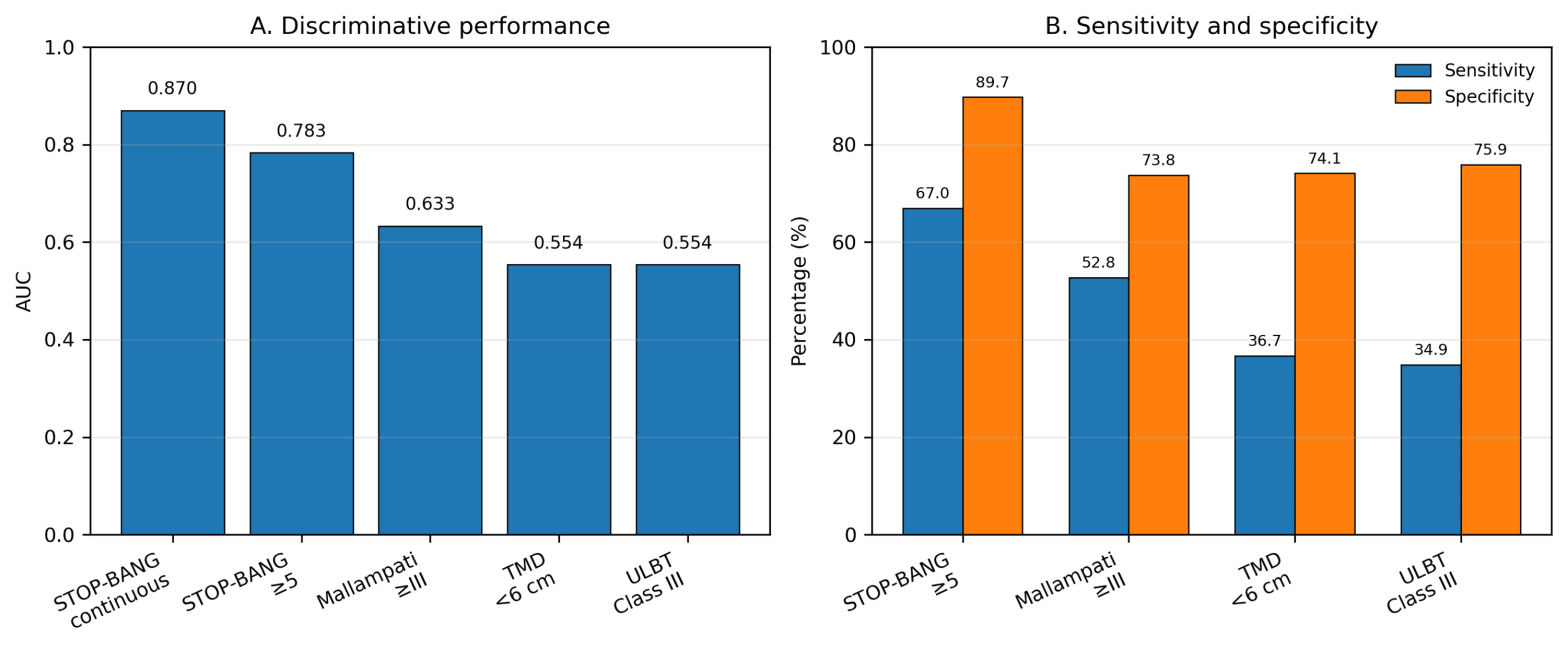
Comparison of predictive performance measures for difficult airway assessment. (A) Comparative AUCs for predicting any airway difficulty using STOP-BANG and preoperative airway assessment tools. (B) Sensitivity and specificity at prespecified thresholds. AUC: area under the curve; TMD: thyromental distance; ULBT: upper lip bite test

Figure 1A shows the comparative AUCs for predicting any airway difficulty across STOP-BANG and the assessed preoperative airway tools, whereas Figure 1B shows the sensitivity and specificity of the main preoperative predictors at prespecified thresholds.

**Figure 2:**
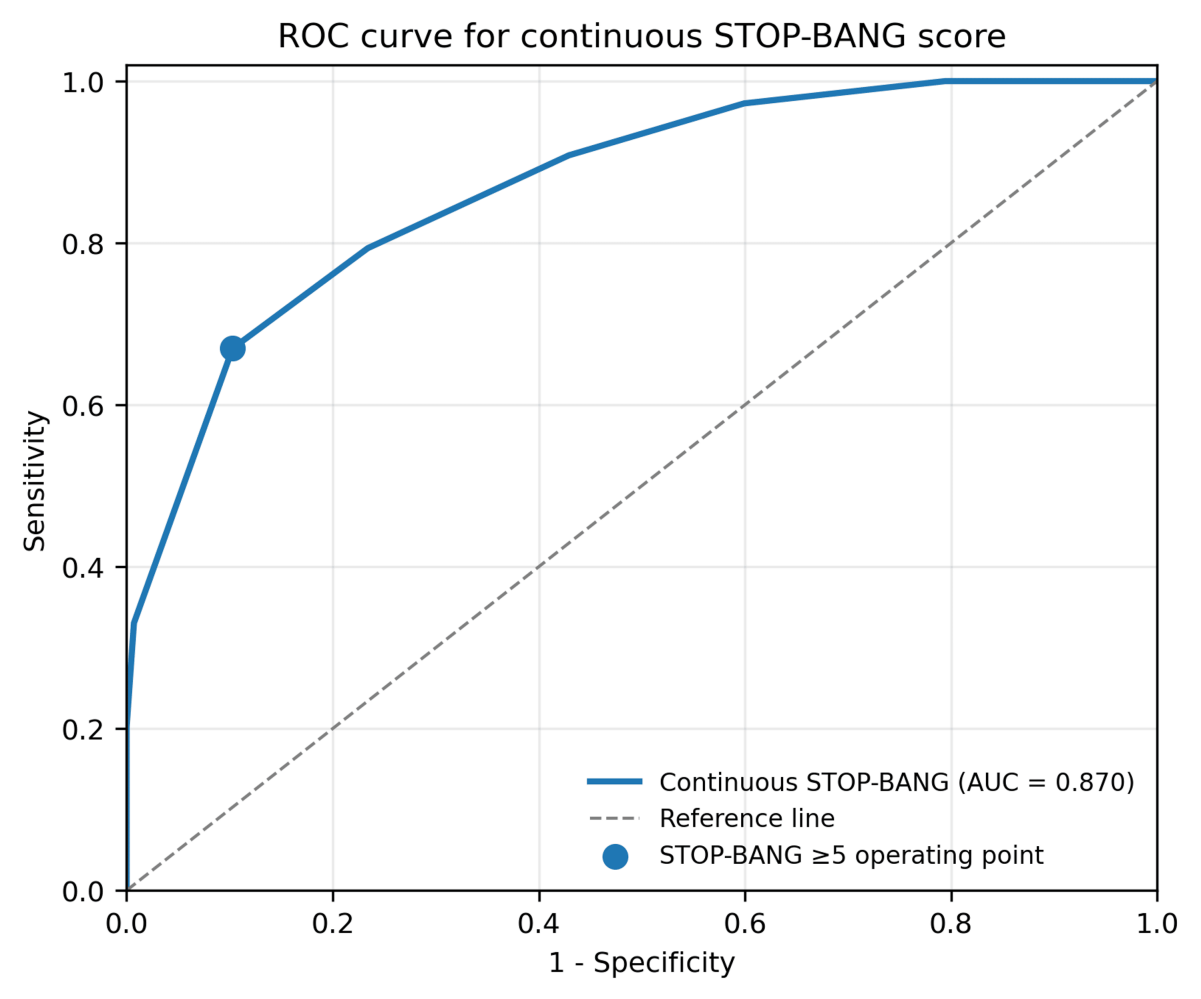
Receiver operating characteristic curve for the continuous STOP-BANG score. ROC curve of the continuous STOP-BANG score for the primary composite endpoint, with the prespecified ≥5 threshold displayed as the operating point.

For the primary composite endpoint, STOP-BANG ≥5 demonstrated a sensitivity of 146/218 (66.97%), specificity of 253/282 (89.72%), positive predictive value of 146/175 (83.43%), negative predictive value of 253/325 (77.85%), positive likelihood ratio of 6.51, and negative likelihood ratio of 0.37. The area under the receiver operating characteristic curve was 0.783 for STOP-BANG ≥5. When analyzed as a continuous score, STOP-BANG demonstrated greater discriminative ability, with an area under the curve of 0.870 for the primary composite endpoint. For difficult intubation alone, the continuous STOP-BANG score had an area under the curve of 0.859. The receiver operating characteristic curve for the continuous STOP-BANG score is shown in Figure 2.

Multivariable analysis

In multivariable logistic regression, STOP-BANG ≥5 was independently associated with any airway difficulty (adjusted odds ratio, 2.84; 95% confidence interval, 1.45-5.57; p = 0.002). STOP-BANG ≥5 was also independently associated with difficult intubation alone (adjusted odds ratio, 2.37; 95% confidence interval, 1.23-4.58; p = 0.010).

Neck circumference >40 cm was strongly associated with both any airway difficulty (adjusted odds ratio, 12.64; 95% confidence interval, 6.83-23.38; p < 0.001) and difficult intubation alone (adjusted odds ratio, 10.63; 95% confidence interval, 5.87-19.27; p < 0.001). Male sex and age >50 years were also independently associated with both outcomes. Body mass index ≥30 kg/m² independently predicted the primary composite endpoint, while its association with difficult intubation alone was borderline. Hypertension was not statistically significant in either model. Multivariable regression results are summarized in Table 3.

**Table 3:** Multivariable logistic regression analysis. aOR: adjusted odds ratio; BMI: body mass index; CI: confidence interval

Predictor	Any airway difficulty aOR (95% CI)	p-value	Difficult intubation aOR (95% CI)	p-value
STOP-BANG ≥5	2.84 (1.45–5.57)	0.002	2.37 (1.23–4.58)	0.010
Neck circumference >40 cm	12.64 (6.83–23.38)	<0.001	10.63 (5.87–19.27)	<0.001
BMI ≥30 kg/m²	1.94 (1.15–3.26)	0.013	1.66 (1.00–2.75)	0.052
Male sex	3.34 (1.90–5.85)	<0.001	3.66 (2.11–6.34)	<0.001
Age >50 years	5.09 (2.80–9.24)	<0.001	5.32 (2.97–9.52)	<0.001
Hypertension	1.75 (0.98–3.12)	0.056	1.62 (0.92–2.82)	0.093

## Discussion

In this single-center prospective observational cohort study of Iraqi adults undergoing elective surgery under general anesthesia, the STOP-BANG questionnaire demonstrated useful predictive performance for difficult airway management. Four main findings were observed. First, a STOP-BANG score of ≥5 identified a clinically relevant high-risk subgroup. Second, this cutoff showed high specificity and positive predictive value for the primary composite endpoint, although sensitivity was moderate. Third, analyzing STOP-BANG as a continuous score provided greater discriminative ability than using a binary cutoff. Finally, STOP-BANG ≥5 remained independently associated with airway difficulty after adjustment for clinically relevant covariates.

These findings are consistent with previous evidence linking OSA, obesity, and upper-airway anatomical characteristics with difficult airway management [5-7]. STOP-BANG has been widely validated as a practical screening tool for OSA in perioperative settings [4,8-12]. The present study extends this evidence by evaluating its predictive utility for airway-management outcomes in an Iraqi surgical population, where local data remain limited.

The stronger performance of the continuous STOP-BANG score suggests that clinically relevant information may be lost when the questionnaire is reduced to a binary classification. This finding supports consideration of the full STOP-BANG score during preoperative airway risk assessment, especially when multiple risk factors are present.

The observed difficult intubation rate of 42.6% was higher than commonly reported rates in studies using narrower definitions. For example, previous work has reported difficult laryngoscopy rates of approximately 22.7% in elective surgical patients when evaluated in relation to bedside airway assessment and direct laryngoscopy [23]. The rate in the present study should be interpreted in light of the broad composite operational definition used, which included an Intubation Difficulty Scale score ≥5, three or more intubation attempts, or Cormack-Lehane grade III or IV. This definition may capture a broader range of airway difficulty than definitions based on a single criterion and should not be interpreted as a rate of failed intubation. The use of direct laryngoscopy with Macintosh blades in a real-world clinical setting may also have contributed to the observed incidence.

Conventional preoperative airway assessment tools, including Mallampati classification, thyromental distance, and upper lip bite test, showed lower discriminative performance than the continuous STOP-BANG score. These findings suggest that STOP-BANG may provide additional value when used alongside traditional airway assessment methods. However, it should not replace comprehensive airway examination, clinical judgment, or preparation for difficult airway management.

The unadjusted cohort comparison showed that patients with STOP-BANG ≥5 had a higher risk of any airway difficulty than patients with STOP-BANG <5. However, the multivariable findings should also be interpreted in context. Several covariates, including age, sex, body mass index, neck circumference, and hypertension, overlap conceptually with STOP-BANG components. These models were therefore intended to evaluate adjusted clinical associations rather than to create a fully independent prediction model. The observed variance inflation factors did not suggest clinically important multicollinearity, but residual overlap between STOP-BANG and its component risk factors remains an important interpretive consideration.

From a practical perspective, STOP-BANG is simple, inexpensive, and easy to apply during preoperative assessment. It may help identify patients who require closer airway planning, particularly in settings where advanced preoperative investigations are not routinely available. Its use may therefore support structured risk stratification before elective surgery under general anesthesia.

Limitations

Several limitations should be acknowledged. This was a single-center prospective observational cohort study conducted in a specific surgical population, which may limit generalizability. External validation was not performed, and the findings should therefore be confirmed in independent multicenter cohorts before broad implementation. A separate screening log was not maintained; therefore, the number and detailed reasons for non-enrollment of patients who did not meet eligibility criteria could not be reported.

External validity should also be interpreted cautiously. Because this study was based on a consecutive convenience sample from a single center, the findings may not be fully generalizable to other hospitals, surgical populations, airway-management protocols, or time periods. Sampling bias, residual confounding, differences in operator experience, and contextual differences in the use of direct laryngoscopy, stylets, neuromuscular blockade, and local airway-management practices may have influenced the observed event rates and predictive performance.

Although intraoperative airway outcomes were assessed using prespecified definitions by experienced anesthesia personnel, and final airway outcome assessment was based on an anesthesiology specialist evaluation, this was a real-world clinical study. Some degree of interobserver variability and ascertainment bias cannot be fully excluded, particularly for clinically assessed outcomes such as mask ventilation grade and laryngoscopic view. The study did not include formal inter-rater reliability testing. However, the use of prespecified definitions, direct laryngoscopy with Macintosh blades, and specialist-confirmed outcome assessment was intended to improve consistency.

Although the enrolled sample size was supported by a diagnostic-accuracy precision justification, a formal a priori sample size calculation was not performed before study initiation. Therefore, the sample size should be considered pragmatic and based on eligible patient availability during the study period. In addition, model calibration was assessed only internally; no external calibration or validation cohort was available. The overlap between STOP-BANG and some multivariable regression covariates should also be considered when interpreting adjusted associations. Because the primary outcome was common, adjusted odds ratios from logistic regression should be interpreted as measures of adjusted association rather than direct estimates of risk ratio.

Despite these limitations, the study has important strengths, including prospective data collection, a relatively large sample size, clearly defined airway outcomes, completely analyzed data, and the use of both discrimination analysis and multivariable modeling.

## Conclusions

The STOP-BANG questionnaire showed clinically useful discrimination for difficult airway management among Iraqi adults undergoing elective surgery under general anesthesia. A STOP-BANG score of ≥5 was associated with a higher risk of any airway difficulty in the unadjusted cohort comparison and remained associated with airway difficulty and difficult intubation in adjusted logistic regression analysis. The continuous STOP-BANG score showed greater discriminative ability than the binary cutoff. These findings support considering STOP-BANG as a practical adjunctive preoperative risk-stratification tool that complements, but does not replace, comprehensive airway assessment and clinical judgment. Further multicenter studies with external validation are needed to confirm these findings in broader populations.

## Figures and Tables

**Figure 3:**
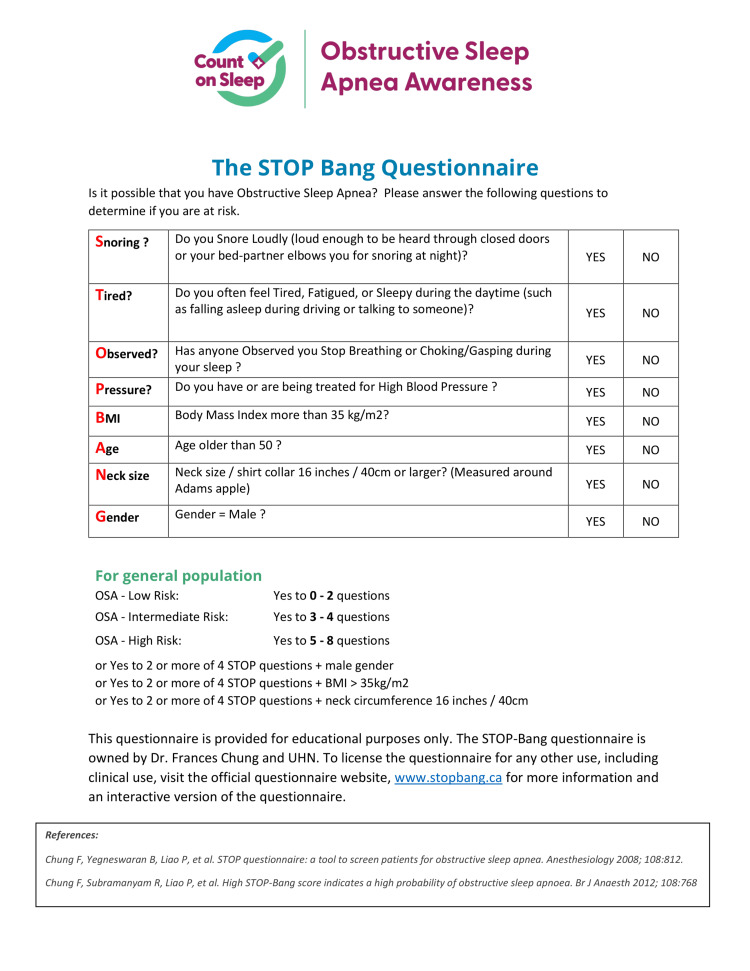
Complete STOP-Bang Questionnaire used for screening obstructive sleep apnea risk in the study participants. The questionnaire consists of eight items (snoring, tiredness, observed apnea, high blood pressure, body mass index, age, neck circumference, and gender) and was used under a non-exclusive academic license from the University Health Network (UHN).
